# Gut microbiota ecology: Biodiversity estimated from hybrid neutral-niche model increases with health status and aging

**DOI:** 10.1371/journal.pone.0237207

**Published:** 2020-10-30

**Authors:** Claudia Sala, Enrico Giampieri, Silvia Vitali, Paolo Garagnani, Daniel Remondini, Armando Bazzani, Claudio Franceschi, Gastone C. Castellani

**Affiliations:** 1 Department of Physics and Astronomy, University of Bologna, Bologna, Italy; 2 Department of Experimental, Diagnostic and Specialty Medicine, University of Bologna, Bologna, Italy; 3 BCAM–Basque Center for Applied Mathematics, Bilbao, Bizkaia, Spain; 4 Lobachevsky State University of Nizhny Novgorod, Nizhny Novgorod, Russia; 5 Galvani Interdepartmental Center, Bologna, Italy; Uniiversity of Padova, ITALY

## Abstract

In this work we propose an index to estimate the gut microbiota biodiversity using a modeling approach with the aim of describing its relationship with health and aging. The gut microbiota, a complex ecosystem that links nutrition and metabolism, has a pervasive effect on all body organs and systems, undergoes profound changes with age and life-style, and substantially contributes to the pathogenesis of age-related diseases. For these reasons, the gut microbiota is a suitable candidate for assessing and quantifying healthy aging, i.e. the capability of individuals to reach an advanced age, avoiding or postponing major age-related diseases. The importance of the gut microbiota in health and aging has been proven to be related not only to its taxonomic composition, but also to its ecological properties, namely its biodiversity. Following an ecological approach, here we intended to characterize the relationship between the gut microbiota biodiversity and healthy aging through the development a parsimonious model of gut microbiota from which biodiversity can be estimated. We analysed publicly available metagenomic data relative to subjects of different ages, countries, nutritional habits and health status and we showed that a hybrid niche-neutral model well describes the observed patterns of bacterial relative abundance. Moreover, starting from such ecological modeling, we derived an estimate of the gut microbiota biodiversity that is consistent with classical indices, while having a higher statistical power. This allowed us to unveil an increase of the gut microbiota biodiversity during aging and to provide a good predictor of health status in old age, dependent on life-style and aging disorders.

## Introduction

The Gut Microbiota (GM) is a complex ecological system composed of a large number of interacting microorganisms with diversified trophic relationships [1]. This inherent complexity has limited the development of predictive models of interaction between the GM and the host. However, following the emerging evidence of its central role in human health and healthy aging [2–5], interest in the GM is considerably increased during the recent years and with it also the number of papers addressing its modelling, both through statistical and mechanistic approaches [6, 7].

While a straightforward theory of GM aging has not yet been designed, various efforts have been made to unveil how GM progresses with age [8] and to develop predictive models of the host biological age based on the GM taxonomic composition [9–11]. Interestingly, besides the specific taxonomic composition, also more global and structural properties of the GM, namely its biodiversity, have been associated with the host life-style, health status and aging [12, 13]. Biodiversity is a fundamental metric commonly used to model and quantify the health status of ecological systems [14, 15]. In ecosystems, in fact, biodiversity has a central role, safeguarding stability and resilience, ensuring sufficient variety of functional traits and species competition, and preventing the predominance of invasive species [16–18]. It is hence not surprising that loss of microbial diversity is one of the most common GM unbalances in human diseases affecting westernized countries [12, 19], accompanied by alterations in GM stability and plasticity [2, 12]. Moreover, a connection between biodiversity and longevity has been hypothesized after the finding of an increase of subdominant species, including opportunistic and allochthonous bacteria but also health-associated taxa, in longevity and extreme longevity [20]. Consistently, loss of GM biodiversity is usually associated with the overgrowth of pathogenic bacteria, fungi and other organisms that may favor excessive energy harvesting from ingested food and inflammatory response [12, 21]. Such disequilibrium has been linked to the emergence of the chronic and systemic low-grade inflammation named inflammaging, associated with morbidity and mortality in elderly people [4, 5, 22–24].

With the aim of investigating the relationship between the GM biodiversity and healthy aging, we consider here an ecological framework. In particular, trading off between the minimal degree of complexity and the maximal power of statistical prediction, we develop an ecological model that describes the GM population stationary distribution and allows to estimate its biodiversity.

Notice that the biodiversity of an ecosystem can also be quantified using more classical approaches that do not rely on the assumption of an underlying dynamical process. There in fact exist various diversity indices that estimate biodiversity starting from the empirical relative abundances of species, such as Shannon [25], Simpson [26], Pielou [27] indices or the Hill’s numbers [28] (see Materials and Methods). However, a major advantage of the modeling approach is that biodiversity is computed directly from the inferred distribution rather than from the relative abundances. This method allows to mitigate the effect of data noise and variability (individual and populations) on the biodiversity index, thus increasing its robustness and general applicability.

Here, we aim to identify a model that well describes the GM ecosystem, to assess whether the biodiversity estimate derived from such model is consistent with the most commonly used classical biodiversity indices and allows to achieve a higher statistical power.

The modelling approach that we consider focuses on characterizing the GM empirical Relative Species Abundance distribution (RSA), a curve that gained a central role in population modelling due to its similarity between different ecological systems. The RSA is constructed counting the number of species with a certain number of individuals and is often well described by long-tailed distributions belonging to the exponential family, such as the Log-Series or the Log-Normal [29].

To explain such regularity various statistical and dynamical models have been proposed [29, 30], and a particularly appealing approach is the one introduced by Volkov et al. [31], that relies on the neutrality [30] assumption. Following this hypothesis, the taxonomic differences among species are neglected and all species are considered to have evolved according to the same dynamics. Specifically, in Volkov’s model the population dynamics of all species included in the ecosystem (i.e. the GM) is ruled by only three parameters: a birth rate (*b*), a death rate (*d*) and an immigration term (*S*) that represents a constant influx of individuals into the population, while inter-species interaction is neglected. The neutrality assumption is certainly an oversimplification of the GM ecosystem dynamics. Nevertheless, this model has been proven to well describe the RSA of various ecosystems [7, 31] and to well characterize their biodiversity [7, 31], while providing an evolutionary explanation of the current RSA configuration.

According to Volkov’s model, indicated here as 1NB model, the RSA is described by a zero-truncated Negative Binomial distribution [31]. As detailed in the Materials and Methods section, such distribution characterizes the probability of having *n* individuals in a species (*P*_*RSA*_*(n)*), and it only depends on the three dynamic parameters *b*, *d* and *S*. Moreover, it holds true for all the species of the ecosystem, and it can be expressed as

$$P_{RSA}(n)=\theta \cdot NB(b,d,S)$$ (1)

to highlight Hubbell’s biodiversity index [30] *θ (see Eq (9) in the* Materials and Methods *section for the definition)*.

The 1NB model fits well the RSA sampled from various ecosystems, including the coral reefs and the GM of several animals [7, 31]. However, incongruence between the neutral 1NB model and empirical data has been previously reported for both ecosystems [7, 32], and suggests that the 1NB model is able to explain part of the GM community structure, but does not well describe the most abundant species that constitute the right tail of the distribution. These findings suggest that along with pure neutral models also hybrid niche-neutral models in which the neutrality assumption is partly relaxed should be taken into account [32]. For these reasons, in this work we try to describe the GM population using the neutral theory but also assuming that we might be observing multiple different ecological niches, each one driven by its own dynamic parameters.

Analysing six publicly available data sets, we show that a model in which two non-interacting niches are considered, well fits the GM data. From this model we derive two biodiversity indices, one for each niche, that overall summarize the GM biodiversity. Finally, we show that such estimates achieve higher statistical power than classical biodiversity indices, in terms of the identification of the relationship between the GM biodiversity and aging and of the discrimation of subjects with different diet and life style or with different health status.

## Results and discussion

### Data sets selection

We selected and analysed publicly available 16S rRNA sequencing data sets related to aging and healthy aging in different populations that either: contained a wide age range of subjects; contained elderly subjects in various health conditions; contained Down Syndrome subjects, a model of accelerated aging [33]. Moreover, a data set consisting of healthy Italians and Tanzanian Hadza hunter-gathers was included to test the descrimintative ability of GM biodiversity (estimated through our modeling approach or using classical indices) under important diet and life-style differences. A brief description of the six selected data sets is provided in the following, while further details about the data sets composition and the pre-processing are reported in the Materials and Methods section and in S1 and S2 Tables.

The analysed data sets are:

- the *ELDERMET* data set [3, 34], that includes 836 samples from 371 Irish elderly (64 to 102 years old) and 13 young (26 to 46 years old) subjects collected at three time points (T0, T1 and T2);
- the *Biagi & Schnorr* data set, composed by the 17 Italian Down Syndrome (DS) adult subjects from Biagi et al. [33] and 16 age-matching healthy Italian adults plus 27 Tanzanian Hadza hunter-gatherers [35];
- the *Odamaki* data set [10], that includes 367 community-dwelling Japanese volunteers between 0 and 104 years old;
- the *Kong* data set [36], that includes 168 Chinese individuals from 24 to 102 years old;
- the *Biagi* data set [20], that includes 69 Italians whose age ranges from 22 to 109 years old;
- the *Bian* data set [37], that includes 1049 Chinese healthy subjects from 3 to 109 years old self-reported as having a personal and family history of extreme health.

### A niche-neutral model for the GM

We modelled the empirical RSA derived from 16S rRNA data considering three possible scenarios. First, we tested pure neutrality by fitting the data with the 1NB model previously introduced. Then, we relaxed the hypothesis of species equivalence considering a hybrid niche-neutral model (2NB model) that assumes the existence of two non-interacting neutral niches (the evolutionary dynamics of each niche is neutral). Finally, we further relaxed the neutral hypothesis contemplating a hybrid niche-neutral model with three niches (3NB model). The 2NB and 3NB models represent a small increase in complexity compared to the pure neutral model [32], by including the possibility of two or more non-interacting niches with different equilibrium properties [29], each summarized by the parameters characterizing the theoretical distribution obtained from the model.

Details on the three models are reported in the Materials and Methods section. According to the selected model, a different population dynamic process is assumed and a different stationary state is reached. Since our aim is not to study the temporal behavior of the GM ecosystem, but rather to exploit the modeling approach to characterize the GM biodiversity at the stationary state, here we focus on the theoretical RSA distribution that is obtained from the three models. Then, we test the accuracy of the model in describing the GM by fitting the empirical RSA obtained from the data with such theoretical distribution.

The stationary distribution of the RSA hypothesized by the 1NB model is given by the Negative Binomial reported in Eq (1). On the other hand, the expected RSA distribution of the 2NB model is a mixture of two zero-truncated Negative Binomials [38], and following the notation of Eq (1), can be written as

$$P_{RSA}\left(n\right)=\alpha \cdot \theta_{1}\cdot NB\left(b_{1},d_{1},S_{1}\right)+\left(1-\alpha \right)\cdot \theta_{2}\cdot NB\left(b_{2},d_{2},S_{2}\right)$$ (2)

where *α* is the mixture coefficient, *b*_*i*_, *d*_*i*_ and *S*_*i*_ are the birth, death, and influx rates of the *i*-th niche (*i* = 1,2), and *θ*_*i*_ is the biodiversity number relative to niche *i*.

Analogously, we assume the stationary RSA distribution for the 3NB model to be a mixture of three zero-truncated Negative Binomials to which 3 biodiversity numbers are associated: *θ*_1_, *θ*_2_ and *θ*_3_. Indicating with *α* and *β* the mixture coefficients, the RSA distribution can be written as

$$P_{RSA}(n)=\alpha \cdot \theta_{1}\cdot NB(b_{1},d_{1},S_{1})+\beta \cdot \theta_{2}\cdot NB(b_{2},d_{2},S_{2})+(1-\alpha -\beta )\cdot \theta_{3}\cdot NB(b_{3},d_{3},S_{3})$$ (3)

Model selection results (S1 Fig) show that the model that better fits the GM RSA is the 2NB. Specifically, in 4 out of 6 data sets (*ELDERMET*, *Kong*, *Biagi* and *Bian*) the selected model is the 2NB, while in the other two data sets (*Biagi & Schnorr* and *Odamaki*) the performances of the three models are mostly comparable. According to the 2NB model, the GM RSA of each sample is described by two Negative Binomials, that respectively account for “rare” and “abundant” species (S2 Fig). Consequently, the two distributions modeling the RSA can be interpreted as referring to rare and abundant species and the RSA distribution can be rewritten as

$$P_{RSA}(n)=\alpha \cdot \theta_{rare}\cdot NB(b_{rare},d_{rare},S_{rare})+(1-\alpha )\cdot \theta_{abundant}\cdot NB(b_{abundant},d_{abundant},S_{abundant})$$ (4)

In this model, the GM biodiversity is given by the combination of two biodiversity numbers, *θ*_*rare*_ and *θ*_*abundant*_, that analogously refer to rare and abundant species. As in the case of the biodiversity index *θ* in the 1NB model (Eq (9)), *θ*_*rare*_ and *θ*_*abundant*_ can be derived from the parameters of the two Negative Binomials and are defined as

$$\theta_{rare}=\frac{N_{obs}}{\left[{\left(1-b_{rare}/d_{rare}\right)}^{-S_{rare}/b_{rare}}-1]\cdot \Gamma (S_{rare}/b_{rare}\right)}$$ (5)

$$\theta_{abundant}=\frac{N_{obs}}{\left[{\left(1-b_{abundant}/d_{abundant}\right)}^{-S_{abundant}/b_{abundant}}-1]\cdot \Gamma (S_{abundant}/b_{abundant}\right)}$$ (6)

In the following subsections we discuss the results obtained from the 2NB model applied to three different biological questions:

1. the relationship between GM biodiversity and aging across several data sets;
2. the discriminative ability of GM biodiversity under important diet and life-style differences and between healthy and unhealthy aging;
3. the ability of GM biodiversity to predict health status in old age.

#### 1. GM biodiversity increases with aging

**Fig 1:**
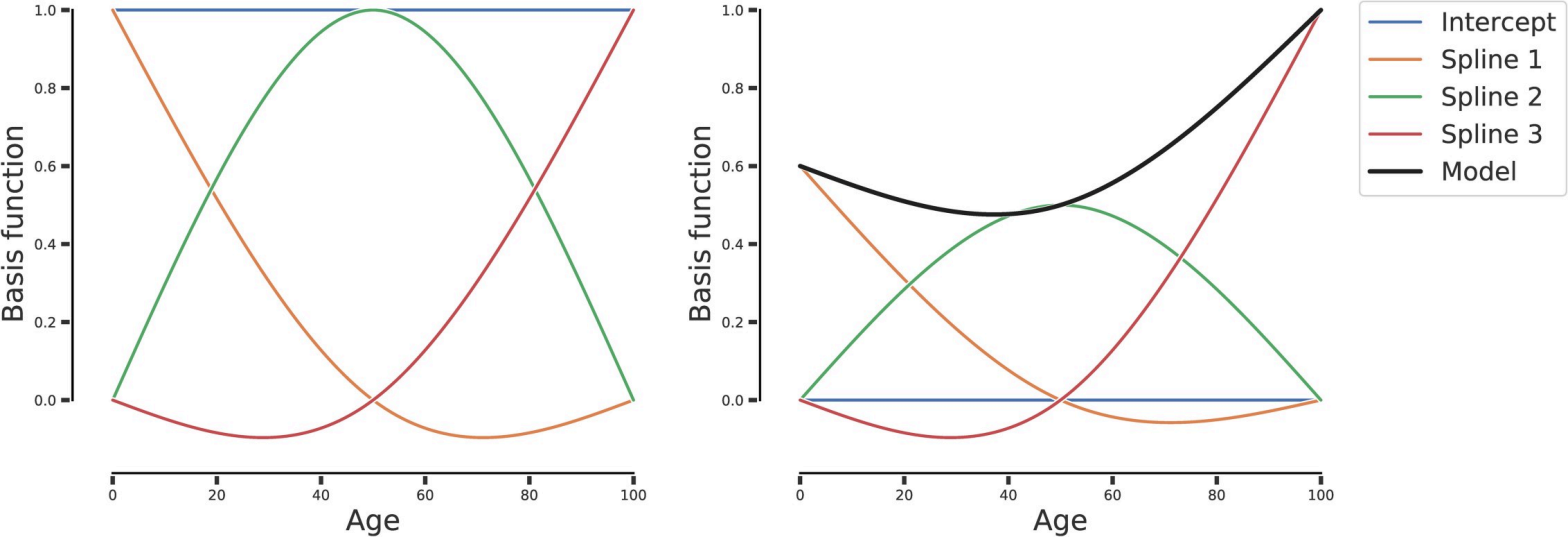
Natural cubic spline model with 3 degrees of freedom. (left) Basis functions for a natural cubic spline with 3 degrees of freedom. (right) Natural cubic spline with 3 degrees of freedom and coefficients equal to: coef(intercept) = 0.0; coef(spline_1_) = 0.6; coef(spline_2_) = 0.5; coef(spline_3_) = 1.0. The black line represents the overall model, while the colored lines are the basis functions rescaled by the model coefficients.

We investigated the general relationship between GM biodiversity, i.e. *θ*_*rare*_ and *θ*_*abundant*_, and aging by fitting a non-linear regression model adjusted for sex and total number of reads, as detailed in the Materials and Methods section. For this purpose, we considered healthy control subjects from all data sets except the Italian controls of Schnorr et al., for which age is not available, and the *ELDERMET* data set samples at times greater than 0, for which young controls are not available. We fitted the model considering samples from all data sets together, while adding the data set of origin as covariate so that to take into account the possible differences related to the individual peculiarities of each data set. As illustrated in Fig 1, the model is given by the weighted sum of three basis functions (splines), that represent the behavior of GM biodiversity in three phases of life: spline_1_ refers to youth years and describes a decrease in biodiversity with age when its coefficient is positive; spline_2_ indicates a high biodiversity in the middle ages but a lower one in young and elderly people when its coefficient is positive; spline_3_ refers to old ages and suggests an increase of biodiversity with age when its coefficient is positive. See Materials and Methods for further details on the spline regression model.

**Fig 2:**
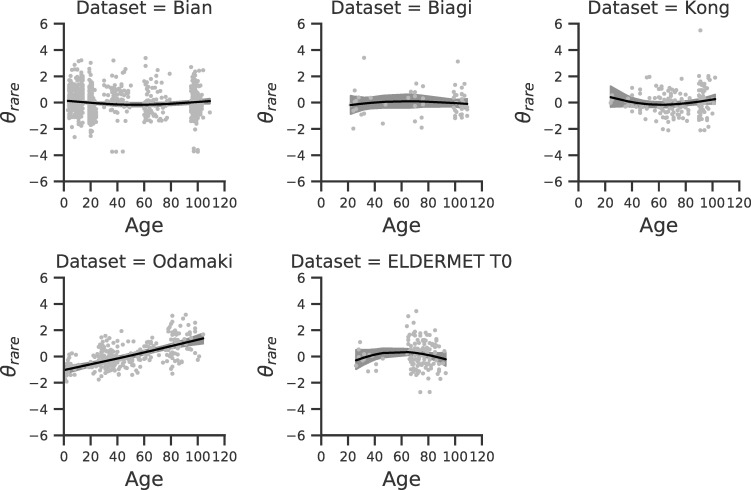
Spline model for *θ*_*rare*_. The splines model corresponding to each individual data set is shown. In each plot, dots correspond to subjects, the x-axis is given by the subject age and the y-axis is the standardized value of *θ*_*rare*_. Black lines and gray shadows represent the data set specific regression lines and confidence intervals.

**Fig 3:**
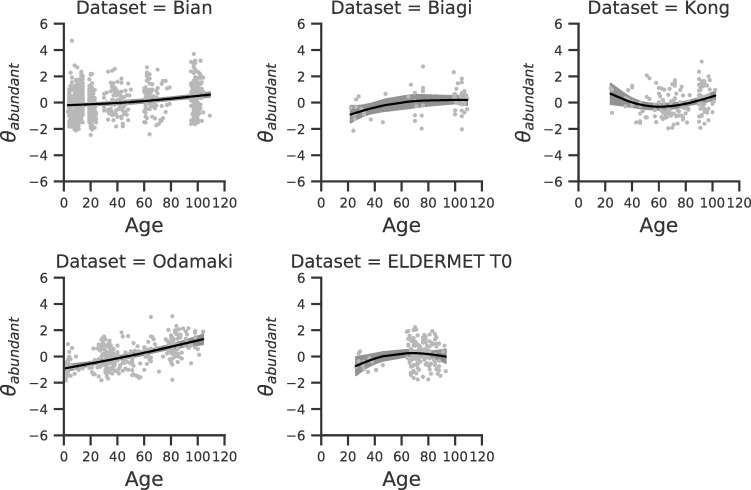
Spline model for *θ*_*abundant*_. The splines model corresponding to each individual data set is shown. In each plot, dots correspond to subjects, the x-axis is given by the subject age and the y-axis is the standardized value of *θ*_*abundant*_. Black lines and gray shadows represent the data set specific regression lines and confidence intervals.

The splines regression model provides a global result that characterizes the average trend of biodiversity with age in all data sets (top block of Table 1), and also data set specific results that describe the deviations of each data set from the average behavior (lower blocks of Table 1, Figs 2 and 3). The global trend results show that on average the coefficient related to spline_1_ is negative, the one of spline_2_ is negligible and the one associated to spline_3_ is positive. This result holds true for both *θ*_*rare*_ and *θ*_*abundant*_ and indicates that biodiversity increases with age in both young and old subjects while staying constant in the middle ages (see Figs 2 and 3, Table 1). For some of the data sets we observe a deviation from the general trend (Table 1). For example, in the *Kong* data set a statistically significant decrease of biodiversity during young age is observed, albeit this could be due to the small number of young subjects.

**Table 1:** Coefficients and t-test p-values for each parameter of the spline regression model.

			θ_rare_		θ_abundant_		Hill_1_		Hill_2_		Pielou		Shannon		Simpson	
			Coef.	P>|t|	Coef.	P>|t|	Coef.	P>|t|	Coef.	P>|t|	Coef.	P>|t|	Coef.	P>|t|	Coef.	P>|t|
	Global model	**Intercept**	-0.104	0.3	0.071	0.465	-0.124	0.205	-0.144	0.147	-0.257	**0.011**	-0.178	0.068	0.159	0.114
		**spline1**	-0.343	0.115	-0.522	**0.014**	-0.708	**0.001**	-0.647	**0.003**	-0.544	**0.014**	-0.708	**0.001**	0.526	**0.017**
		**spline2**	0.015	0.9	0.007	0.954	0.096	0.416	0.14	0.242	0.172	0.161	0.151	0.2	-0.157	0.196
		**spline3**	0.225	**0.024**	0.586	**<0.001**	0.488	**<0.001**	0.363	**<0.001**	0.115	0.256	0.379	**<0.001**	-0.209	**0.038**
Data set specific deviations from the global model	*Bian*	**Intercept**	0.049	0.643	-0.128	0.21	0.024	0.818	-0.001	0.992	0.19	0.075	0.068	0.507	-0.047	0.658
		**spline1**	0.447	**0.046**	0.153	0.482	0.586	**0.008**	0.695	**0.002**	0.593	**0.009**	0.684	**0.002**	-0.648	**0.004**
		**spline2**	-0.236	0.081	-0.139	0.288	-0.47	**<0.001**	-0.575	**<0.001**	-0.651	**<0.001**	-0.618	**<0.001**	0.66	**<0.001**
		**spline3**	-0.163	0.153	-0.141	0.202	-0.092	0.411	-0.122	0.282	0.247	**0.033**	0.002	0.988	-0.06	0.605
	*Biagi*	**Intercept**	-0.117	0.515	-0.36	**0.04**	-0.404	**0.022**	-0.33	0.065	-0.349	0.057	-0.377	**0.033**	0.304	0.095
		**spline1**	-0.047	0.926	-0.702	0.15	-1.07	**0.03**	-1.01	**0.042**	-1.31	**0.01**	-1.097	**0.025**	1.011	**0.046**
		**spline2**	0.151	0.594	0.278	0.312	0.713	**0.01**	0.716	**0.011**	0.755	**0.009**	0.636	**0.022**	-0.705	**0.014**
		**spline3**	-0.221	0.244	0.064	0.728	-0.047	0.802	-0.036	0.85	0.207	0.284	0.085	0.648	-0.002	0.993
	*Kong*	**Intercept**	0.377	0.096	0.694	**0.002**	0.339	0.127	0.198	0.379	0.234	0.309	0.399	0.072	-0.184	0.422
		**spline1**	1.119	**0.038**	1.943	**<0.001**	1.068	**0.043**	0.591	0.269	0.528	0.335	1.129	**0.032**	-0.536	0.325
		**spline2**	-0.506	**0.035**	-0.821	**<0.001**	-0.368	0.117	-0.232	0.329	-0.219	0.368	-0.425	0.069	0.178	0.461
		**spline3**	-0.235	0.23	-0.429	**0.024**	-0.36	0.061	-0.161	0.406	-0.075	0.707	-0.304	0.111	0.174	0.378
	*Odamaki*	**Intercept**	0.138	0.213	0.283	**0.008**	0.233	**0.031**	0.215	0.05	0.05	0.657	0.119	0.269	0.018	0.872
		**spline1**	-0.851	**<0.001**	-0.466	**0.041**	-0.373	0.104	-0.255	0.273	0.117	0.622	-0.475	**0.038**	0.427	0.071
		**spline2**	-0.125	0.405	-0.032	0.824	0.12	0.414	0.174	0.242	0.309	**0.043**	0.373	**0.011**	-0.527	**0.001**
		**spline3**	1.114	**<0.001**	0.782	**<0.001**	0.486	**0.002**	0.296	0.068	-0.376	**0.023**	0.221	0.165	0.118	0.475
	*ELDERMET T0*	**Intercept**	-0.446	0.105	-0.489	0.067	-0.192	0.476	-0.082	0.764	-0.125	0.653	-0.21	0.434	-0.091	0.742
		**spline1**	-0.668	0.224	-0.928	0.082	-0.21	0.697	-0.021	0.969	0.072	0.897	-0.241	0.653	-0.254	0.648
		**spline2**	0.716	**0.033**	0.715	**0.028**	0.006	0.987	-0.083	0.802	-0.194	0.568	0.035	0.916	0.393	0.246
		**spline3**	-0.494	0.082	-0.276	0.317	0.012	0.965	0.023	0.936	-0.003	0.991	-0.003	0.99	-0.23	0.421
	Covariates	**Sex**	0.228	**<0.001**	0.086	0.067	0.254	**<0.001**	0.259	**<0.001**	0.28	**<0.001**	0.287	**<0.001**	-0.264	**<0.001**
		**NumOfReads**	-0.008	0.844	0.271	**<0.001**	0.234	**<0.001**	0.262	**<0.001**	0.134	**0.001**	0.23	**<0.001**	-0.204	**<0.001**

The upper block of parameters refers to the global behavior (Global model), while in the following blocks show how each data set deviates from that trend. P-values are bold if < 0.05. When statistically significantly different from zero, cells with coefficients and corresponding p-values of the Global model are shaded red (if positive) or blue (if negative). Due to its opposite trend, inverted colors are used for results relative to the *Simpson* index.

We remark that the data set specific deviations from the global trend are related to the effect of all the variables that differ between one data set and the others, including sequencing technology, 16S targeted region, pre-processing of data, population ethnicity, and all the other data set specific unknown sources of variation. Indeed, the global model is not affected by such sources of variation, nor by the different sample size of the data sets.

In the model, in fact, the categorical variable relative to the data set of origin was treated as a Contrast coding. This means that the results for the general model are defined as the grand mean (average of the means) of the effects, and they are therefore independent from the numerosity of each individual data set. This allowed us to include data sets with different sample size without distorting the results.

To corroborate our results, we fitted the splines regression model considering five among the most common classical biodiversity indices: *Hill*_*1*_ [28], *Hill*_*2*_ [28], *Pielou* [27], *Shannon* [25] and *Simpson* [26] index. The mathematical definitions of these indices are reported in the Materials and Methods section. Here, we only recall that for all indices but *Simpson*, higher values correspond to higher biodiversity. Table 1 shows that the increase of GM biodiversity with age is detected not only by *θ*_*rare*_ and *θ*_*abundant*_, but also by all other indices, with the only exception of *Pielou*, that does not identify a statistically significant increase of biodiversity for old subjects. Notice that *θ*_*rare*_ does not detect the increase of GM biodiversity with age in young subjects. However, according to the 2NB model *θ*_*rare*_ and *θ*_*abundant*_ describe the biodiversity of two different niche of the GM ecosystem and should therefore be taken into account simultaniously.

#### 2. GM biodiversity and healthy aging

We tested the discriminative ability of the biodiversity indices *θ*_*rare*_ and *θ*_*abundant*_ considering the *Biagi & Schnorr* data set, that includes individuals from 3 groups: i) Italian healthy subjects [35] (Healthy Italian), ii) age-matched Italian DS subjects (DS Italian), that represent a model of accelerated aging [33]; and iii) Hadza hunter-gatherers of Tanzania, that are known to have a life-style that is beneficial for the GM [35] (Healthy Hadza). Biodiversity data were adjusted for the total number of reads before performing the hypothesis tests, while sex balance among the groups was guaranteed by the original authors [33, 35].

**Fig 4:**
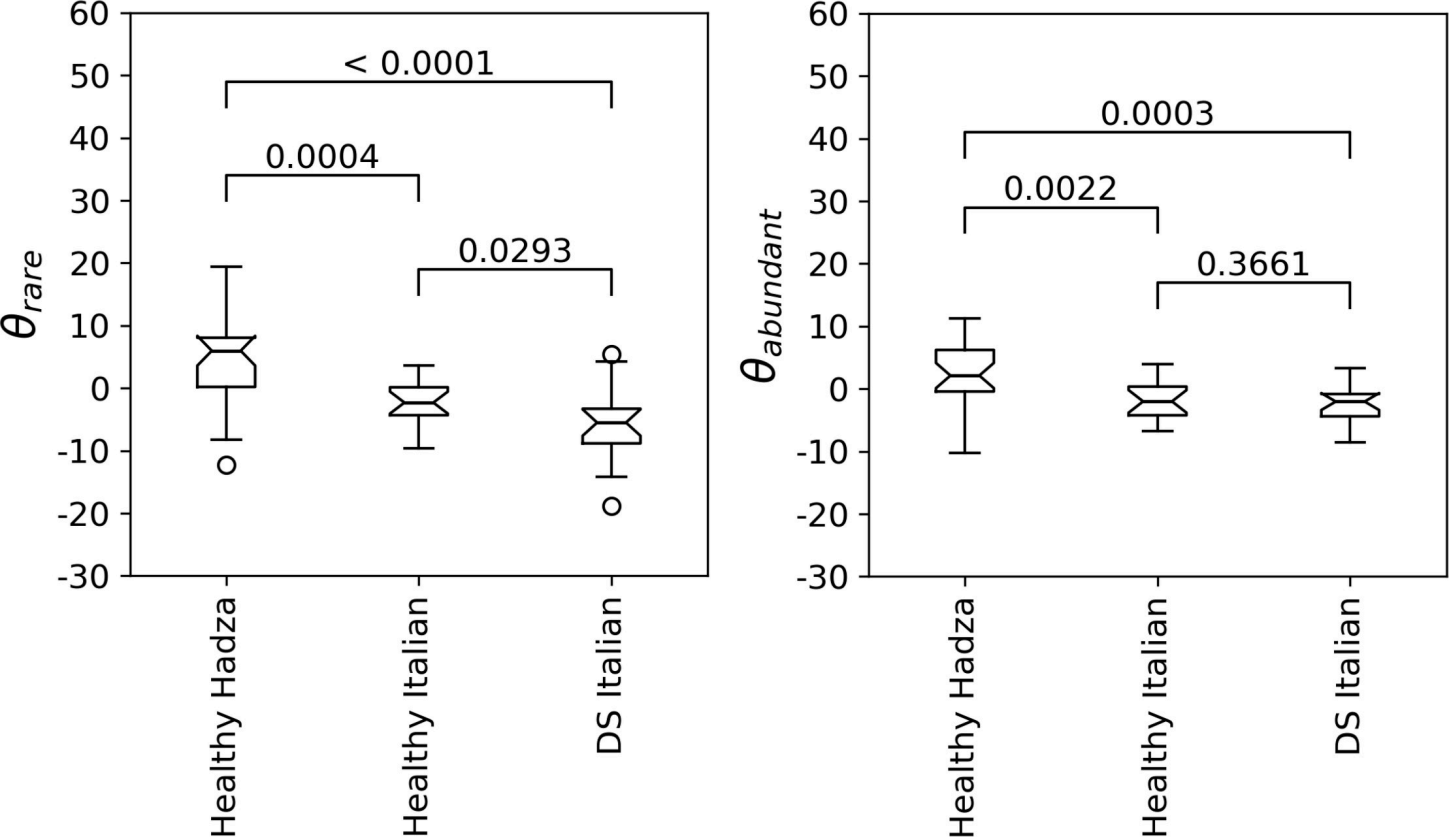
GM biodiversity and health status. Box-plots representing the distribution of GM biodiversity (*θ*_*rare*_ on the left and *θ*_*abundant*_ on the right) in the three groups of Healhty Hadza, Healthy Italian controls and DS Italian subjects. Mann-Whitney U-test p-values are reported for each pairwise comparison. Both biodiversity indices were adjusted for the total number of reads before computing the box-plots and hypothesis tests.

In agreement with previous findings [35] and with the results obtained using other classical biodiversity indices (S8 Fig), the GM biodiversity turns out to be statistically significantly higher in the Healthy Hadza compared to the Healthy Italian control group, both when considering *θ*_*rare*_ (Mann-Whitney U-test p-value = 0.0004) and *θ*_*abundant*_ (Mann-Whitney U-test p-value = 0.0022), as shown in Fig 4. This result confirms the ability of *θ*_*rare*_ and *θ*_*abundant*_ to detect differences in GM biodiversity in the presence of important diet and life-style differences.

Regarding the comparison between Healthy Italian controls and DS Italian subjects, instead, the differences detected in the original paper [33] or using classical biodiversity indices such as *Pielou*, *Shannon* or *Simspon* (S8 Fig) are negligible. However, when estimating the GM biodiversity from the 2NB model, we detect a statistically significant decrease of GM biodiversity (*θ*_*rare*_) in DS subjects compared to the control group (Mann-Whitney U-test p-value = 0.0293), and consistent results are obtained with the *Hill*_*1*_ and *Hill*_*2*_ numbers (Mann-Whitney U-test p-value = 0.043 and 0.047, respectively).

Overall, the outcome of the pairwise comparisons between the three groups of the *Biagi&Schorr* data set suggests that the estimate of biodiversity derived from the 2NB model is consistent with classical indices, while having a higher statistical power (Fig 4 and S8 Fig). When comparing Healthy Italian and Healthy Hadza, in fact, the test based on *θ*_*rare*_ achieves the lowest p-value, followed by *θ*_*abundant*_; when comparing Healthy Hadza and DS Italian, the smallest p-value is obtained with *θ*_*rare*_, followed by *Shannon* and by *Hill*_*1*_ and *θ*_*abundant*_, tied for third place; and when comparing Healthy Italian and DS Italian subjects, the smallest p-value is obtained with *θ*_*rare*_, followed by *Hill*_*1*_ and *Hill*_*2*_, while none of the other indices detects a statistical significant difference.

Such increase in statistical power allows us to obtain an interseting result that is not obvious when using classical statistical indices: DS subjects have a lower GM biodiversity than age-matched controls. Notice that DS is associated with accelerated aging [33] and that the global trend of the splines regression model reported in the previous section indicates a general increase of GM biodiversity with age. This suggests that the increase of GM biodiversity with age that we observed in such model is related to healthy aging and that a decrease of GM biodiversity is associated with unhealthy phenotypes rather than with a slower aging.

**Fig 5:**
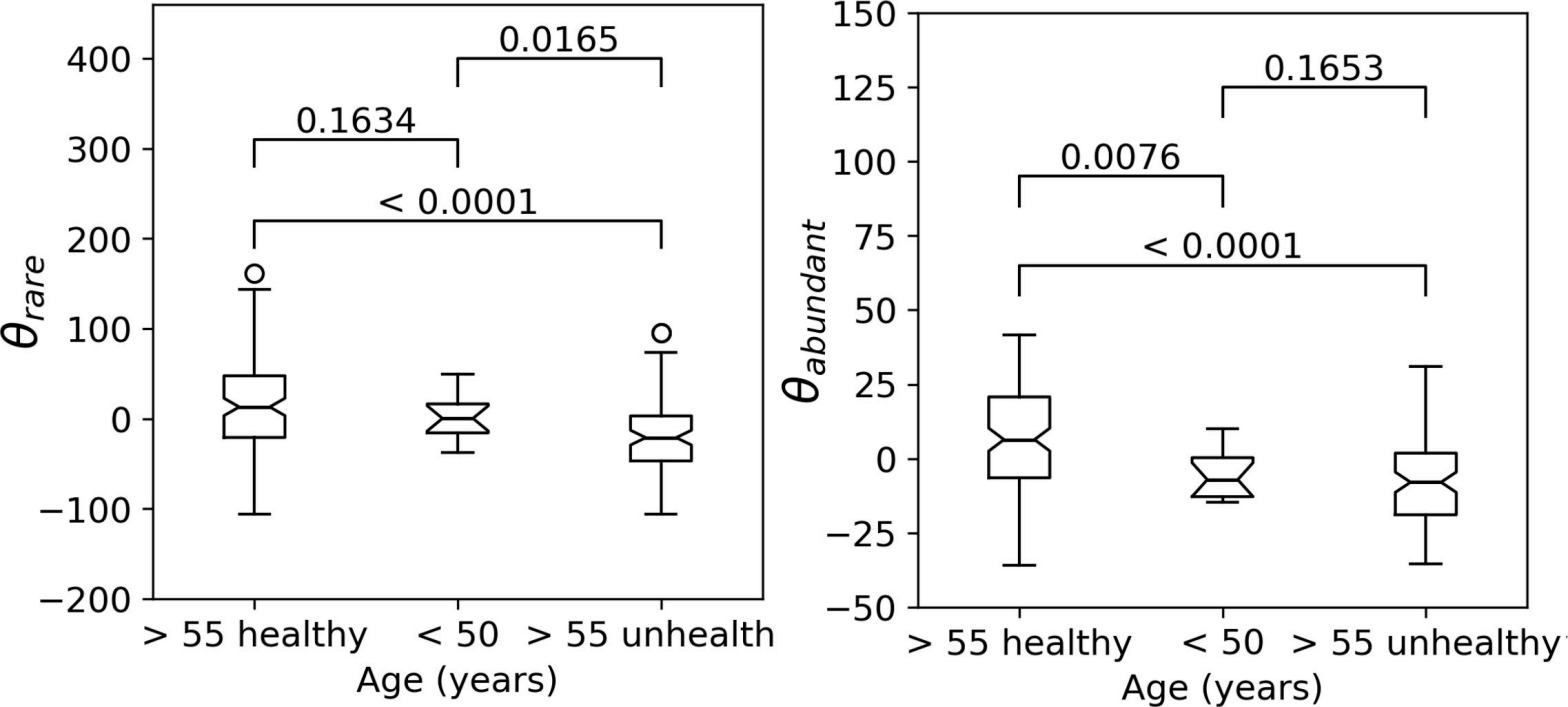
GM biodiversity and healthy aging status. Box-plots representing the distribution of GM biodiversity (*θ*_*rare*_ on the left and *θ*_*abundant*_ on the right) in the healthy elderly, young controls and unhealthy elderly subjects from the *ELDERMET* data set at T0. Mann-Whitney U-test p-values are reported for each pairwise comparison. Both biodiversity indices were adjusted for sex and total number of reads before computing the box-plots and hypothesis tests.

This interpretation was corroborated by the investigation of the GM biodiversity of the healthy and unhealthy elderly subjects from the *ELDERMET* data set at T0 (see the Materials and Methods section for details on the classification of subjects). Fig 5 shows that while healthy elderly subjects have a statistically significantly higher GM biodiversity than young controls, elderly subjects classified as unhealthy have a statistically significantly lower GM biodiversity than both healthy elderly and young controls.

The analysis of the *ELDERMET T0* data set also allowed us to confirm the results about the consistence and statistical power of the estimate of biodiversity obtained from the 2NB model. When performing the hypothesis tests based on classical biodiversity indices, in fact, we obtain results that are in agreement with those revealed by *θ*_*rare*_ and *θ*_*abundant*_ (S9 Fig), even if not all classical indices are able to detect all the differences between the three groups (S9 Fig). Moreover, when ranking biodiversity indices according to the p-values, it turns out that the most significant results are obtained with *θ*_*rare*_ or *θ*_*abundant*_ for all comparisons, except that of healthy versus unhealthy elderly subjects, in which *Simpson* obtaines the smallest p-value (0.0028), followed by *θ*_*rare*_ (p-value = 0.0165), while none of the other indices detects a stastically significant difference (Fig 5 and S9 Fig). This supports the hypothesis that estimating biodiversity through the 2NB model provides more statistical power.

### GM biodiversity predicts health status in old age

We assessed the ability of the GM biodiversity estimated by the 2NB model to predict the health status in the elderly considering the *ELDERMET T0* data set. To this aim, we fitted a linear regression model between health status and GM biodiversity adjusted for sex and total number of reads, and we evaluated the results obtained with GM biodiversity estimated by *θ*_*rare*_ and *θ*_*abundant*_, as well as using other traditional biodiversity scores (*Hill*_*1*_, *Hill*_*2*_, *Simpson*, *Shannon* and *Pielou* index). See the Materials and Methods section for details.

Results show that *θ*_*rare*_ and *θ*_*abundant*_ have good predictive capability toward health status (AUC of ROC = 0.701), with *θ*_*rare*_ showing the best performances (Table 2). For all the other classical biodiversity indices, the obtained predictive accuracy is always lower (Table 2) and their AUC of ROC is comparable to the one obtained when only sex and the total number of reads are used as predictive variables (AUC of ROC = 0.644).

**Table 2:** AUC of ROC of the predictive models of health status in old age.

Biodiversity index	AUC of ROC	
	Without aging parameters	With aging parameters
*θ*_*rare*_ + *θ*_*abundant*_	0.701	0.76
*θ*_*rare*_	0.724	0.77
*θ*_*abundant*_	0.701	0.766
Hill1	0.636	0.757
Hill2	0.648	0.763
Simpson	0.645	0.745
Shannon	0.649	0.759
Pielou	0.645	0.751
Only sex + # of reads	0.644	0.759

All models were adjusted for sex and total number of reads (# of reads). The last row refers to models in which GM biodiversity was not included. The two columns “With aging parameters” and “Without aging parameters” refers to models in which calf circumference, BMI and the inflammatory markers IL-6, IL-8, IL-10, TNFa and CRP were or were not included as explanatory variables.

To further evaluate the relationship between GM biodiversity and healthy aging, we compared the performance of the model in which health status is explained by GM biodiversity with a model in which the explanatory variables are a set of parameters traditionally associated with the health status of elderly people and available for the *ELDERMET T0* data set: calf circumference, BMI and the inflammatory markers IL-6, IL-8, IL-10, TNFa and CRP. The relationships between such parameters, health status and healthy aging are shown in the correlation heat map of S6 Fig.

As expected, the model based on the traditional aging parameters (adjusted for sex and total number of reads) has good predictive performances towards health status (AUC of ROC = 0.759). Such performances are better than those obtained by any biodiversity index (Table 2). Finally, when considering a model in which both GM biodiversity and the aging parameters are included, the increase in performances is limited (Table 2, column “With aging parameters”), and an improvement is only obtained when biodiversity is quantified by *θ*_*rare*_ and *θ*_*abundant*_ (S7 Fig, Table 2) or by the *Hill*_*2*_ number (Table 2), suggesting again that estimating biodiversity with the 2NB models allows to achieve higher statistical power.

## Conclusions

We presented here an ecological model capable of describing the GM ecosystem. Our model is a hybrid niche-neutral model composed of two neutral and non-interacting populations that respectively include the least abundant (rare) and the most abundant (abundant) bacterial species. This approach is not an attempt to accurately describe the exact structure of the GM, but rather to assess whether some of the properties of the GM and its relationship with aging could be described with a minimalistic model.

Fitting the model to the data allows to infer a biodiversity index for each of the two populations, given by Hubbell’s diversity index [30, 31] and named here *θ*_*rare*_ and *θ*_*abundant*_.

The code to derive *θ*_*rare*_ and *θ*_*abundant*_ from the OTU counts table is available at https://github.com/UniboDIFABiophysics/AlphaDiversityPublic.

Analysing six publicly available data sets, we showed that our estimate of the GM biodiversity is coherent with those obtained with classical indices. However, *θ*_*rare*_ and *θ*_*abundant*_ appeared to have a higher statistical power when analysing the pattern of GM biodiversity with age and the relationship between biodiversity and diet or health status.

Our results unveiled that GM biodiversity increases with age, with the exception of the middle ages, in which it remains constant. This finding was verified using *θ*_*rare*_ and *θ*_*abundant*_ as biodiversity estimates, but also confirmed with other classical biodiversity indices (*Hill*_*1*_, *Hill*_*2*_, *Pielou*, *Shannon* and *Simpson* index).

The trend of GM biodiversity across the lifespan is still a matter of debate, especially in the elderly [39, 40]. Some studies suggest that GM biodiversity increases with age in healthy elderly [10, 36, 37], in agreement with our result, while others do not reveal any change of GM biodiversity throughout such age range [41–43].

Moreover, even when the attention is focused on extreme aging and the GM biodiveristy of centenarians is analysed, results are discordant [36, 39, 42]. Some studies observe a decrease of GM biodiversity in centenarians compared to younger elderly [42], while others detect an increase [36].

Such controversies are possibly due to the high variability of data between and within data sets. Investigating the global trend of five different data sets, here we aimed to achieve a higher statistical power and, as already mentioned, we found a general increase of GM biodiversity with age. Our results, however, also highlighted some discrepancies between data sets and did not allow to draw conclusions on narrow age ranges, specifically for centenarians. Hence, further studies will be needed to validate our result and to clarify how GM biodiversity varies with age and how it behaves in extreme aging.

The analysis of three case studies, allowed us to conclude that GM biodiversity is also related to the host health status and to healthy aging.

We found that GM biodiversity of subjects with Down Syndrome, a model of accelerated aging [31], is lower than that of healthy controls from the same population and matched for age and sex. Moreover, we found that healthy elderly have a higher GM biodiversity compared to young subjects, as expected from the result on the increase of GM biodviersity with age. However, unhealthy elderly have a biodiversity that is lower than both young subjects and healthy elderly, corroborating the hypothesis that an increase of GM biodiversity is related to healthy aging rather than to chronological aging alone.

This result is in agreement with previous findings in which a decrease of GM biodiveristy was found to be associated with frailty [41], biological age [43] and hospitalization [3], and pointed us towards the development of a predictive model of health status in old age based on GM biodiversity. Analysing healthy and unhealhty elderly from the ELDERMET data set, we found that *θ*_*rare*_ and *θ*_*abundant*_ are good predictors of healthy aging (AUC of ROC = 0.701). On the contrary, other biodiversity indices did not show any improve predictive ability compared to sex and total number of reads alone. This confirms the greater statistical power of *θ*_*rare*_ and *θ*_*abundant*_ indices compared to the traditional ones to address healthy aging.

Overall, during the analysis of the three case studies we noticed that, while results were often confirmed using classical biodiversity indices, not all of them were able to detect all the differences or had the same statistical power. Our results suggest that estimating GM biodiversity through the ecological modeling that we proposed allows to derive biodiversity indices that are appropriate, i.e. consistent with other classical biodiversity indices, but enable to achieve higher statistical power and to unveil differences that would otherwise be masked by the intrinsic noise of the GM measurements.

## Materials and methods

### Data sets description, pre-processing and OTUs computation

We considered 6 publicly available data sets containing 16S rRNA gene sequencing data of subjects for which the information about age and health status was available. We choose these data sets as they contained either a wide age range, data about aging-related diseases or data related to subjects with important diet and life-style differences. In the following, we describe the main features of each data set and the pre-processing pipeline that was used to obtain *de novo* Operational Taxonomic Units (OTUs). When processed sequencing data or OTUs were available, these were considered and eventually further processed. Notice that the pre-processing pipeline was adapted to each data set according to the choices performed by the original authors. Since the modelling and statistical analysis were performed separately for each data set, here the aim of the pre-processing is in fact to obtain clean data and not to obtain OTUs that are comparable between data sets. The main characteristics in terms of sequencing and bioinformatic processing are summarized in S1 Table. The data sets characteristics in terms of age, sex, health status and numerosity of individuals included in the statistical analyses are summarized in S2 Table.

i) *ELDERMET*: The ELDERMET data set [3, 34], includes 836 samples from 371 elderly (64–102 years old) and 13 young (26–46 years old) Irish subjects. Faecal samples were collected at 3 time points, approximately 3 months apart, that we will refer to as T0, T1 and T2. DNA was extracted from faecal samples, and sequence reads from 16S rRNA gene V4 amplicons were sequenced on a 454 Genome Sequencer FLX Titanium platform (Roche Diagnostics and Beckman Coulter Genomics) according to the manufacturer’s sequencing protocol. DNA sequences (fastq files) are available on the Sequence Read Archive under BioProject PRJNA283106. Besides age, available personal and clinical information for the elderly people include gender, antibiotics usage, Body Mass Index (BMI), calf circumference, residence setting, Mini Nutritional Assessment (MNA), Healthy Food Diversity index (HeFD), Functional Independence Measures (FIM), Mini Mental State Exam (MMSE), Barthel score, and values for interleukin (IL)-6, IL-8, IL-10 and Tumour Necrosis Factor (TNF)-α. Sequencing reads were filtered using fastx-toolkit [44] according to the following criteria: read length not shorter than 150 bp and not longer than 350 bp; no ambiguous bases (Ns); quality score higher than 25 in at least 50% of the read. OTUs were then obtained by clustering reads at 97% similarity using the UPARSE pipeline [45].

ii) *Biagi & Schnorr*: The data sets from Biagi et al. [33] and Schnorr et al. [35] include 17 Italian Down Syndrome (DS) persons and 16 age-matched Italian healthy young adults (20–40 years old) who adhered to the standard Mediterranean diet. The study from Schnorr et al. [35] also includes 27 Hadza hunter-gatherers (8–70 years old), whose diet is mainly based on meat, honey, baobab, berries and tubers. For all subjects, age is available but the match between the subject age and 16S rRNA data is not available. 16S rRNA gene V4 amplicons were sequenced on a 454 Genome Sequencer FLX Titanium platform (Roche Diagnostics and Beckman Coulter Genomics) according to the manufacturer’s sequencing protocol. DNA sequences (fasta files) are respectively available on MG-RAST under the project ids mgp10557 and mgp7058. Sequencing reads were filtered using mothur [46] according to the following criteria: read length not shorter than 150 bp and not longer than 350 bp; no ambiguous bases (Ns). Quality filter was not applied because fastq files were not available. Then, OTUs were obtained by clustering reads at 97% of similarity using the UPARSE pipeline [45].

iii) *Odamaki*: Data from Odamaki et al. [10] include 367 community-dwelling Japanese (0–104 years old). 16S rRNA gene V3-V4 amplicons were sequenced using an Illumina MiSeq instrument (Illumina, Inc., San Diego, CA, USA) with a MiSeq v3 Reagent Kit.

DNA sequences (fastq files) are available in DDBJ under accession number DRA004160.

Paired-end sequencing reads were merged using usearch and filtered with usearch using the following criteria: read length not shorter than 150 bp; average quality score greater than 25. OTUs were obtained by clustering sequencing reads at 97% following the UPARSE pipeline [45].

iv) *Kong*: Data from Kong et al. [36] include 168 Chinese healthy subjects (24–102 years old) from Dujiangyan and Ya'an, Sichuan province. 16S rRNA gene V4-V5 amplicons were sequenced on an Illumina MiSeq sequencer (Illumina, Inc., San Diego, CA, USA) using a 2×250 bp paired protocol. DNA sequences (merged paired-end fastq files) are available on the Sequence Read Archive under the accession number SRP076167. Sequencing reads were filtered using mothur [46] according to the following criteria: read length not shorter than 150 bp and not longer than 400 bp; no ambiguous bases (Ns); no homopolymers longer than 8 bp, average quality score greater than 25. OTUs were obtained by clustering sequencing reads at 97% following the UPARSE pipeline [45].

v) *Biagi*: Data from Biagi et al. [20] include 69 Italian healthy subjects (22–109 years old) from Emilia-Romagna. 16S rRNA gene V3-V4 amplicons were sequenced on an Illumina MiSeq sequencer (Illumina, Inc., San Diego, CA, USA) using a 2×300 bp paired end protocol. DNA sequences (fasta files) are available on MG-RAST under the project id 17761. Since specific filters and parameters applied for the pre-processing of the reads are not detailed in the original paper, we used the filters of the *Kong* data set, adapting the parameters to the different read length. Hence, sequencing reads were filtered using mothur [46] according to the following criteria: read length not shorter than 400 bp and not longer than 500 bp; no ambiguous bases (Ns); no homopolymers longer than 8 bp. Quality filter was not applied because fastq files were not available. OTUs were obtained by clustering sequencing reads at 97% following the UPARSE pipeline [45].

vi) *Bian*: Data from Bian et al. [37] include 1125 Chinese healthy subjects (3–109 years old) self-reported as having a personal and family history of extreme health. Among these 212 are young soldiers (19–24) who passed the standard military entrance medical examination, and whose grandparents lived to be at least 85 years. 16S rRNA gene V4 amplicons were sequenced on an Illumina MiSeq sequencer (Illumina, Inc., San Diego, CA, USA) using a 2×300 bp paired end protocol. OTUs across all samples were obtained from the Supplementary Information of the original paper.

### Modeling of the Relative Species Abundance distribution (RSA)

According to Volkov’s model [31], the population dynamics of all species included in the ecosystem is a birth-death process with a further constant influx. The dynamics is hence ruled by three parameters: a birth rate (*b*), a death rate (*d*) and an immigration term (*S*) that represents a density dependent constant influx of individuals into the population, and can be expressed by the deterministic equation

$$\frac{dn}{dt}=b\cdot n-d\cdot n+S$$ (7)

Volkov treats this model in the framework of the Chemical Master Equation and proves that under these assumptions the probability distribution of the RSA is expected to be the zero-truncated Negative Binomial

$$P_{RSA}(n)=\frac{N_{obs}}{1-{\left(1-b/d\right)}^{S/b}}\frac{{\left(1-b/d\right)}^{S/b}}{\Gamma (S/b)}\frac{{\left(b/d\right)}^{n}}{n!}\Gamma (n+S/b)$$ (8)

where *N*_*obs*_ refers to the total number of observed species and *Γ* is the gamma function. Biodiversity can be estimated using the Hubbell biodiversity index [30, 31], defined as

$$\theta =\frac{N_{obs}}{\left[{\left(1-b/d\right)}^{-S/b}-1]\cdot \Gamma (S/b\right)}$$ (9)

We modelled the empirical RSA derived from 16S rRNA data considering three possible scenarios. First, we tested pure neutrality by fitting the data with the 1NB model (Eq (8)). Then, we relaxed the hypothesis of species equivalence considering a hybrid niche-neutral model (2NB model) that assumes the existence of two non-interacting neutral niches (the evolutionary dynamics of each niche is neutral).

Finally, we further relaxed the neutral hypothesis contemplating a hybrid niche-neutral model with three niches (3NB model).

The mathematical aspects of the 2NB model are detailed in Bazzani et al. [38] and show that the stationary RSA distribution is a mixture of two zero-truncated Negative Binomials

$$P_{RSA}(n)=\alpha \cdot \theta_{1}\frac{{\left(b_{1}/d_{1}\right)}^{n}}{n!}\Gamma (n+S_{1}/b_{1})+(1-\alpha )\cdot \theta_{2}\frac{{\left(b_{2}/d_{2}\right)}^{n}}{n!}\Gamma (n+S_{2}/b_{2})$$ (10)

where α is the mixture coefficient, *b*_*i*_, *d*_*i*_ and *S*_*i*_ are the birth, death, and influx rates of the *i*-th niche (*i* = 1,2), and *θ*_*i*_ is the biodiversity number relative to niche *i*, and is equivalent to the one in Eq (9).

Analogously, we assume the stationary RSA distribution for the 3NB model to be a mixture of three zero-truncated Negative Binomials (Eq (11)) to which 3 biodiversity numbers are associated: *θ*_1_, *θ*_2_ and *θ*_3_.

$$P_{RSA}(n)=\alpha \cdot \theta_{1}\frac{{\left(b_{1}/d_{1}\right)}^{n}}{n!}\Gamma (n+S_{1}/b_{1})+\beta \cdot \theta_{2}\frac{{\left(b_{2}/d_{2}\right)}^{n}}{n!}\Gamma (n+S_{2}/b_{2})+(1-\alpha -\beta )\cdot \theta_{3}\frac{{\left(b_{3}/d_{3}\right)}^{n}}{n!}\Gamma (n+S_{3}/b_{3})$$ (11)

### Model fitting and model selection

Empirical RSA distributions were computed by counting the number of OTUs with a certain number of individuals. The 1NB, 2NB and 3NB models were fitted to the data using a custom implementation of the Approximate Bayesian Computation (ABC) rejection algorithm.

Assuming a degree of similarity across samples, the goal was to implement a hierarchical model to estimate the parameters of the model, allowing the estimate from the general population to inform about the plausible values of each individual sample.To facilitate the algorithm convergence, we implemented this method with a two-step approach. In the first step, we assigned uninformative prior distributions to the model parameters and we fitted the model for each sample using the ABC algorithm detailed below. Then, for each data set, we constructed the posterior distributions of the model parameters pooling the accepted parameters of all samples belonging to that data set and fitting them with either a Beta distribution (mixture coefficients) or a Gamma distribution (all the other parameters). Notice that, in general, the number of accepted parameters was highly variable from sample to sample. For some samples the number of parameters accepted in the first step was very small or null. For other samples, however, thousands of parameters were identified as acceptable. For this reason, we chose to limit the number of accepted parameters obtained from each sample to 5. This allowed to avoid high unbalances in the number of parameters derived from each sample, as well as to improve the computational efficency of the posterior fitting. Finally, the posterior distributions obtained in the first step were used as data set-specific prior distributions and the ABC algorithm was run again to obtain the final model estimates (second step). In both fitting steps, for 10^7^ times we randomly sampled a set of parameters from their prior distributions and we simulated a number of data equal to the number of OTUs according to the selected model (1NB, 2NB or 3NB). Then, we evaluated the accordance between the simulated and the empirical RSA comparing the two Preston’s plots. This choice was made to reduce the numerical issues related to the sparsity of the data in the heavy tailed distributions [30]. Specifically, each set of parameters was accepted if both the conditions described in the following were satisfied. First, we set the maximum acceptable absolute difference between data and simulation counts in each bin to 30% of the data counts. Secondly, we constructed a variant of the chi-squared test that was appropriate for the comparison of the two observed samples (real and simulated data). Our null hypothesis, here, is that the difference between the observed values for each bin of the Preston’s plot is distributed as a Skellam’s distribution (the difference between two statistically independent random variables, each Poisson-distributed with respective expected values *μ*_*1*_ and *μ*_*2*_). Given that this distribution converges to the Normal distribution, we can use a variant of the chi-squared test obtained by summing the standardized Skellam’s distributions of each bin of the Preston’s plot:

$$\chi^{2}=\sum_{i=1}^{N}\frac{{\left(\mu_{i1}-\mu_{i2}\right)}^{2}}{\left(\mu_{i1}+\mu_{i2}\right)}$$ (12)

where *i* indicates the *i*-th bin of the Preston’s plot.

The number of degrees of freedom was set equal to the number of non-zero bins and the set of simulated parameters was accepted when the probability that the simulated and true bins of the Preston’s plot come from the same distribution was higher than the probability that they did not, i.e. when the chi-squared cumulative distribution at *χ*^2^ was lower than 0.5. This criteria was designed according the samples acceptance criteria in ABC. Finally, for each sample, the posterior distributions of the parameters were computed considering all accepted set of parameters.

Model selection was achieved comparing the posterior probability of each model, estimated computing the ratio between the number of accepted parameters sets over the total number of simulated parameters (10^7^), obtaining a median number of accepted parameters of ~2000 samples (acceptance rate of ~2·10^−4^, relative error in the rate estimation of 2%). Specifically, for each pair of models we computed the logarithm of the ratio of the two posterior probabilities of each sample.

This method computes an approximation of the Logarithmic Bayes Factor, defined as the log ratio of the probabilities that each model is the actual true model, without incorporating prior beliefs about the plausibility of each mode [47].

This method is akin to a Bayesian equivalent of the likelihood ratio tests such as BIC (Bayesian Information Criterion) and AIC (Akaike Information Criterion) [48], but including an implicit penalization for the number of parameters that each model possesses and the shape of the prior for each parameter: wider priors (encoding for less certain parameters) cause a greater penalization than narrower ones (encoding for more information available about them).

All the methods were implemented in python 3.6.8 [49] using the following libraries: patsy [50], pandas [51], numpy [52], scipy [53], matplotlib [54], seaborn [55], pymc [56]. The code to perform the parameter estimation through ABC starting from the OTU abundances table is available on a github repository at https://github.com/UniboDIFABiophysics/AlphaDiversityPublic.

### Classical biodiversity indices

Results obtained with *θ*_*rare*_ and *θ*_*abundant*_ were compared to those obtained when biodiversity was computed using classical biodiversity indices that are based on the empirical relative abundance of OTUs. Specifically, we considered *Shannon* [25], *Pielou* [27], *Hill*_*1*_ [28], *Simpson* [26] and *Hill*_*2*_ [28] indices, whose definitions are reported in the following.

Let us call p_i_ the proportion of individuals belonging to the i-th OTUs and S the total number of OTUs. Then,

1. *Shannon* index (entropy) is defined as *H* = −∑*p*_*i*_⋅*ln*(*p*_*i*_)
2. *Pielou* index (eveness) is defined as *J* = *H*/*H*_*max*_, were *H*_*max*_ is the maximum possible value of *Shannon* index *H*, that is achieved when *p*_*i*_ *= 1/S* for each *i*, *i*.*e*. $H_{max}=-\sum \frac{1}{S}\cdot ln\left(\frac{1}{S}\right)$
3. *Hill*_*1*_ number (Hill number of order 1) is defined as *Hill*_1_ = *exp*(*H*)
4. *Simspon* index is defined as $\lambda =\sum p_{i}^{2}$. Notice that differently from the other indices *Simpson* index decreases with biodiversity.
5. *Hill*_*2*_ number (Hill number of order 2) is defined as ${Hill}_{2}=\frac{1}{\lambda }$

### Spline regression: Modeling the trend of GM biodiversity with aging

The general relationship between the GM biodiversity indices, *θ*_*rare*_ and *θ*_*abundant*_, and aging was investigated by fitting a natural cubic splines model [57] adjusted for sex (0 = male, 1 = female) and standardized total number of reads, using the library patsy [50] in python 3.6.8 [49]. See the following paragraph “Notes on splines regression” for details.

Here, we considered the healthy control subjects from all the data sets except the Italian controls of Schnorr et al., for which age was not available, and the *ELDERMET* data sets at times greater than 0, for which young controls were not available. Samples with outlier total number of reads (z-score > 4) were removed prior the analysis. These included 6 elderly subjects from the *ELDERMET* study at T0 data set, 8 subjects from the *Bian* data set, 4 subjects from the *Odamaki* data set, and 1 subject from the *Kong* data set.

The GM biodiversity estimates, *θ*_*rare*_ and *θ*_*abundant*_, were analysed separately. After standardizing the diversity indices within each data set, we fitted the natural cubic spline model considering the data set to which the samples belong as confounding variable.

The number of degrees of freedom was set to 3 based on the Akaike Information Criteria (AIC) and the Bayesian Information Criteria (BIC), as shown in S3 Fig.

The spline regression model was also computed considering classical biodiversity indices, i.e. the *Pielou*, *Shannon*, *Simpson*, *Hill*_*1*_, and *Hill*_*2*_ indices.

### Notes on splines regression

Splines regression models (also referred to as Generalized Additive Models [57]) allow to describe non-linear behaviors in the data using the framework of linear regression, where the outcome variable is described as the linear combination of generic, non linear functions of the independent variables. While traditionally this was performed combining functions such as squares and cubes of the data, the spline regression uses a different set of basis function, one of which is always the average value of the outcome variable, usually referred to as the intercept, albeit improperly. The other functions are chosen from a family of functions depending on the desidered properties. In the case of biological functions, a common approach is to fit with a polynomial (in this case of 3rd order) a subset of the data, while imposing 4 conditions: continuity of value, continuity of derivative of first order, continuity of the derivative of second order, and the overall function at the boundaries is linear. These are usually referred to as natural cubic splines. The number of knots (the junction points between different parts of the data set) were chosen using a cross-validation approach, using the AIC criterion as the chosen metric. Due to the constrains required for this functions to create a smooth fit, they can exhibit small fluctuations outside the original domain, such as those that can be seen in spline 1 and 3 in Fig 1.

### Classification and prediction of health status in old age

Elderly samples from the *ELDERMET* data set were classified as healthy or unhealthy according to the FIM score [58], Barthel index [59] and MMSE score [60] and to the residence setting. In particular, for each subject, a score of 1 was assigned for each of the following conditions when they were true: Barthel score ≥ 15 [58], MMSE score ≥ 24 [60], FIM score ≥ 100 [58] and residence setting in Community or Day-Hospital. Then, the subject was classified as healthy if the sum of the scores was ≥ 3 or unhealthy otherwise.

Considering the elderly subjects from the *ELDERMET* study at T0 that were not outliers according to the total number of reads, we computed a predictive model for health status in old age based on GM biodiversity. After imputing missing data with the Multiple Imputation by Chained Equations (MICE) method [61] and standardizing the covariates, we computed a Principal Component Analysis (PCA) based on the elderly physical and cognitive state scores, (FIM score, Barthel index and MMSE score) and the residence setting (S5 Fig). Most of the variance (91.9%) was explained by the first Principal Component (PC[0]) that was hence considered as the measure of healthy aging in the following predictive model.

The predictive model of health status in old age based on GM biodiversity was computed as a Leave-One-Out cross validated linear regression and various possible GM biodiversity measurements were adopted as explanatory variables: besides *θ*_*rare*_ and *θ*_*abundant*_, we also considered the *Pielou*, *Shannon*, *Simpson*, *Hill*_*1*_, and *Hill*_*2*_ indices.

After dichotomizing the subjects true and predicted health status in healthy/unhealthy, we computed the Area Under the Receiver Operating Characteristic Curve (AUC of ROC). The models were all adjusted for sex and standardized number of reads and computed both with and without the addition of calf circumference, BMI and the inflammatory markers IL-6, IL-8, IL-10, TNFa and CRP as furhter explanatory variables.

## Supporting information

S1 Fig Model selection. Logarithm of the ratio of the posterior probabilities of model 2NB and 1NB (left), of model 2NB and 3NB (center) and of model 1NB and 3NB (right). Each posterior probability has been increased by a small factor (10^−7^) to avoid infinite value in the log-ratio. When comparing models 2NB and 1NB (left), data below the red line are those for which the 2NB model is selected rather than the 1NB. When comparing models 2NB and 3NB (center), data above the red line are those for which the 2NB model is selected rather than the 3NB. When comparing models 1NB and 3NB (right), data above the red line are those for which the 1NB model is selected rather than the 3NB.
(EPS)

S2 Fig Example of Preston plot and fit. Preston plot of the sample SRR3679961 of the Kong data set (gray histogram). The box-plots represent the predicted values obtained over the ABC iterations. The magenta and blue lines are two Negative Binomial distributions obtained using the median of the parameters of the ABC iterations respectively concerning “rare” and “abundant” species.
(EPS)

S3 Fig AIC and BIC of natural cubic spline model. AIC and BIC (y-axis) of the natural cubic spline model adjsuted for sex and scaled total number of reads, when varying the number of degrees of freedom (x-axis). Results for *θ*_*rare*_ are shown on the left and those for *θ*_*abundant*_ on the right. For both AIC and BIC, lower values are better.
(EPS)

S4 Fig Biodiversity variability within subjects. For each subject of the *ELDERMET* data set for which data at the 3 time points were available, we plot the values of *θ*_*rare*_ and *θ*_*abundant*_. Subjects are sorted according to the minimum average biodiversity. The plots show that the between-samples variability of both *θ*_*rare*_ and *θ*_*abundant*_ is higher than the within-sample variability.
(EPS)

S5 Fig Principal Component Analysis of the *ELDERMET T0* health state. Representation of the *ELDERMET* elderly subjects (at T0) in the space defined by the first two components of the PCA computed using the FIM score, the Barthel index, the MMSE score and residence setting as covariates. Subjects are colored according to the healthy and unhealthy classification that was defined based on the same parameters used for the PCA.
(EPS)

S6 Fig Relationship between the variables of the *ELDERMET T0* data set. Heat map of the Pearson’s r correlation matrix computed for the *ELDERMET* data set at T0.
(EPS)

S7 Fig Results of the predictive model of health status in old age. Empirical and predicted values of the first PC. The plotted model was evaluated using as explanatory variables *θ*_*rare*_ and *θ*_*abundant*_, plus calf circumference, BMI and the inflammatory markers IL-6, IL-8, IL-10, TNFa and CRP, and adjusting for sex and standardized total number of reads. AUC of ROC was computed after dichotomizing the subjects health status in healthy/unhealthy. Bisector represents perfect prediction. AUC of ROC results for the alternative predictive models are reported in S2 Table.
(EPS)

S8 Fig DS results obtained with classical biodiversity indices. (EPS)

S9 Fig ELDERMET results obtained with classical biodiversity indices. (EPS)

S1 Table Summary of the data sets. For each data set, we summarize the number of individuals for each decade of age, divided by sex and health status. Here, we considered only subjects that were included in the statistical analysis.
(XLSX)

S2 Table Summary of sequencing and bioinformatic processing. Title. For each data set, we report the main sequencing and bioinformatic methodologies applied to samples, including (when available) sequencing platform and technology, reagent version, target region, algorithm used to merge reads (for paired reads), read filtering rules and OTU calculation.
(XLSX)
